# Supplementary material for: Inflammatory factors and risk of lung adenocarcinoma: a Mendelian randomization study mediated by blood metabolites
Source: Front Endocrinol (Lausanne). 2024 Aug 27;15:1446863. doi: 10.3389/fendo.2024.1446863 (PMC11384989; doi:10.3389/fendo.2024.1446863)
Supplement: Supplementary file 1 [file DataSheet1.zip › supplementary files/S2/GCST90200335/sensitivity-analysis.pdf]

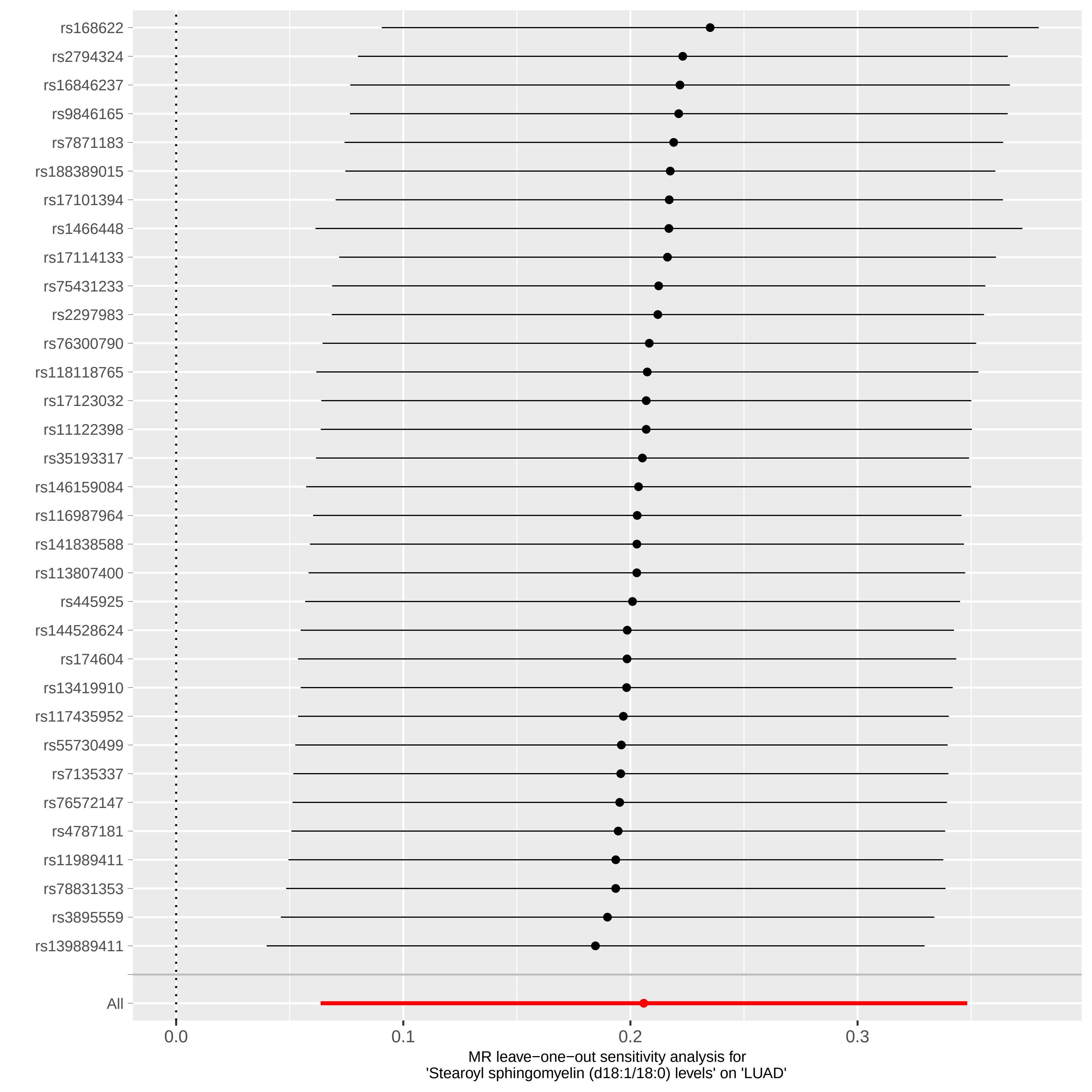

rs168622
rs2794324
rs16846237
rs9846165
rs7871183
rs188389015
rs17101394
rs1466448
rs17114133
rs75431233
rs2297983
rs76300790
rs118118765
rs17123032
rs11122398
rs35193317
rs146159084
rs116987964
rs141838588
rs113807400
rs445925
rs144528624
rs174604
rs13419910
rs117435952
rs55730499
rs7135337
rs76572147
rs4787181
rs11989411
rs78831353
rs3895559
rs139889411
All
0.0
0.1
0.2
0.3
MR leave−one−out sensitivity analysis for
'Stearoyl sphingomyelin (d18:1/18:0) levels' on 'LUAD'
